# What constitutes important change in individual PROMIS-10 global health items in patients with high-impact chronic pain: a qualitative interview study

**DOI:** 10.1007/s11136-026-04164-5

**Published:** 2026-01-22

**Authors:** Emily Sophia Madley, Daniel Broholm, Sophie Lykkegaard Ravn, Henrik Bjarke Vaegter

**Affiliations:** 1 Pain Research Group, Pain Center, University Hospital Odense, C, Heden 7-9, Indgang 200, 5000 Odense, Denmark; 2 Department of Clinical Research, Faculty of Health Sciences, University of Southern Denmark, Odense, Denmark; 3 Pain Center Middelfart, University Hospital of Southern Denmark, Middelfart, Denmark; 4 Department of Psychology, Faculty of Health Sciences, University of Southern Denmark, Odense, Denmark; 5 Specialized Hospital for Polio and Accident Victims, Roedovre, Denmark

**Keywords:** Chronic pain, PROMIS-10 Global health, Important change, Qualitative interviews, Quality of life

## Abstract

**Purpose:**

Health-related quality of life is a key outcome for patients with high-impact chronic pain, and the PROMIS-10 Global Health questionnaire is widely used to assess it. This study examined what patients perceive as important changes in PROMIS-10 Global Health items by 1) 1) examining how much each item should change to be perceived as important, and 2) exploring why and how these changes were considered important.

**Methods:**

Individual semi-structured interviews with 17 participants with high-impact chronic pain were conducted. Analysis for objective one involved determining item-level thresholds for important change by aggregating participants’ reported change scores for each item. Analysis for objective two involved thematic analysis to explore how and why such changes were perceived as important.

**Results:**

For items rated on a 5-point Likert scale, change scores perceived as important ranged from a mean of 1.09 (median = 1) for item 1 (general health) to a mean of 1.84 (median = 2) for item 8r (fatigue). For item 7r (pain intensity), rated on a 0–10 scale, a mean change score of 4.36 (median = 4.25) was considered important. Thematic analysis identified one to two themes per item, reflecting perceived important improvement across physical, mental, and social health.

**Conclusion:**

This is the first qualitative study to explore what patients with high-impact chronic pain perceive as important changes in PROMIS-10 Global Health items. Findings reflect important improvements across physical, mental, and social health, highlighting the value of patient perspectives in clinical use. Further qualitative research is needed to enhance interpretation and inform clinical applications.

**Supplementary Information:**

The online version contains supplementary material available at 10.1007/s11136-026-04164-5.

## Abstract (unstructured)

Patients living with high-impact chronic pain often face serious challenges in their daily lives, including poor physical and mental health. For these patients, improving health-related quality of life is a key goal. To measure this, healthcare providers and researchers often use the PROMIS-10 Global Health questionnaire. However, little is known about what patients with high-impact chronic pain consider an important change in their responses to this questionnaire. This study aimed to explore how much each item in the PROMIS-10 Global Health needs to change for patients to see it as important, and why these changes are important to them. We interviewed 17 patients with high-impact chronic pain. They were asked how much each item would need to improve for them to perceive the changes as important and why these changes were considered important. Patients reported that a 1- to 2-point improvement on items rated from 1 to 5 was important. For pain intensity, rated from 0 to 10, a reduction of 4.36 points was seen as important. These changes were considered important because patients believed they would lead to better physical functioning, improved mental wellbeing, and more participation in social life. The results highlight the importance of listening to patients' perspectives when using health questionnaires, so that treatment and research can better reflect what truly matters to them.

**Supplementary Information:**

The online version contains supplementary material available at 10.1007/s11136-026-04164-5.

## Introduction

Chronic pain is a prevalent and disabling health condition affecting approximately 20% of the global population [1]. Among these individuals, a substantial subgroup (5–15%) lives with high-impact chronic pain (HICP) [2], characterized by ongoing pain and significant interference with daily activities lasting at least three months [3]. HICP is associated with increased psychological distress, cognitive difficulties, and heightened need for health and social care [3]. Patients with HICP consistently report poor health-related quality of life (HRQOL) [4, 5], making it a key outcome in clinical care and research [6].

HRQOL is a multidimensional concept that encompasses individuals’ perceptions of their physical, mental, and social well-being [7]. In the context of HICP, assessing HRQOL is particularly important to evaluate treatment outcomes and guide efforts to enhance care [6]. To evaluate HRQOL in a valid and standardized manner, patient-reported outcome measures (PROMs) are commonly used [7]. One such widely applied instrument in chronic pain research and clinical practice used to measure HRQOL is the Patient-Reported Outcomes Measurement Information System Global Health Survey (PROMIS-10 GH) [7, 8]. PROMIS-10 GH provides a comprehensive assessment of health, including dimensions such as physical, mental, and social functioning [8].

When interpreting change scores in PROMs, such as PROMIS-10 GH, establishing thresholds for important change is essential [9]. Although PROMIS-10 GH is frequently used to evaluate intervention outcomes [7, 8], little is known regarding what constitutes important change from the patient’s perspective. Existing quantitative studies have defined minimally important change (MIC) thresholds at the domain level across chronic pain populations [10, 11], but these vary by population and methodology (e.g., anchor- vs. distribution-based) and rely on statistical rather than patient-perspective definitions. Consequently, they do not provide insight into how and why specific item-level changes are perceived as important in daily life [12]. Since PROMIS-10 GH is also applied at item-level to support clinical dialogue and shared decision-making in clinical practice [13], and PROMIS items are used in Computer Adaptive Testing (CAT) where single-item interpretation is central [14], understanding important change at item-level from a patient perspective is increasingly important.

To ensure PROM change scores reflect patient priorities, qualitative research on perceived important change at item-level is essential [9, 15]. Qualitative methods are increasingly recognized for their value in identifying what constitutes an important change and why such change matters from the patient’s perspective [15, 16]. They can provide insight into how much change is needed for it to be perceived as important, why this change is important, and what it implies for daily life and functioning [9]. Such knowledge is crucial for interpreting treatment effectiveness and guiding clinical decision-making [9, 15]. However, no previous qualitative studies have examined what constitutes important change in PROMIS-10 GH at the item-level, or why and how these changes are important for people with HICP. To address this gap, we conducted a qualitative interview study with patients with HICP to explore: (1) how much responses in each item should change on their respective response scale for the patients to perceive it as important and (2) why and how patients perceive these response changes as important in the context of their lived experiences.

## Materials and methods

### Approach and concepts

This study adopts an individual-level interpretation of health outcomes approach using a consensus-based method based on patients’ judgments [17]. The applied concept is MIC, defined as the smallest within-person change over time perceived as important [9].

### Ethics

This study was preregistered on the Open Science Framework (10.17605/OSF.IO/5724P) and registered at Odense University Hospital on the 24th of October 2023 (journal no. 23/48829). According to Danish legislation, formal ethical approval was not required, as the study was based solely on patient-reported data and interviews without testing an intervention or conducting an experiment [18]. The study complied with the Declaration of Helsinki [19] and the General Data Protection Regulation; all participants provided written informed consent, and data were pseudonymized and securely stored [20].

### PROMIS-10 GH

The PROMIS-10 GH v1.2 measures self-reported physical and mental health across ten items covering physical, psychological, and social aspects of health [21]. Items are rated on a 5-point Likert scale, except for item 7r (pain intensity), which uses an 11-point numeric rating scale from 0 (no pain) to 10 (worst imaginable pain) [22]. Two summary domain scores (physical and mental health) between 4 and 20 can be calculated, with higher scores indicating better HRQOL [22]. The physical health domain includes items 3 (physical function), 6 (ability to carry out everyday physical activities), 8r (fatigue), and 7r (pain intensity) (reversed scored). The mental health domain includes items 2 (quality of life), 4 (mental health), 5 (social activities and relationships), and 10r (emotional problems). Item 1 (general health) and item 9r (satisfaction with social roles and activities) are not included in the domain scores. Sum scores can be converted to T-scores standardized to the U.S. general population (mean = 50, SD = 10), ranging from 16.2 to 67.7 for physical and 21.2–67.6 for mental health [21–23].

### Recruitment and participants

Participants were recruited from the waiting lists of two interdisciplinary pain centers in Denmark: Odense University Hospital [24] and the University Hospital of Southern Denmark [25]. Eligible participants were Danish proficient adults (≥ 18 years) living with HICP referred for treatment at one of the centers. As part of routine care at the pain centers, patients complete a clinical questionnaire via the electronic pain registry, PainData [5]. For this study, an additional item was included in the questionnaire, allowing patients to give consent to be contacted regarding participation. Those who agreed were subsequently contacted by ESM, who provided information about the study. To ensure a diverse sample, purposive sampling was applied based on characteristics such as age, ethnicity, and gender [26]. Patients who were eligible and interested in participating were invited to an interview at one of the pain centers. Recruitment and interviews were conducted on a rolling basis. Following only qualitative research recommendations, the target sample size was set at 15–20 participants [27].

### Data collection

Data were collected by ESM, a female master’s student in Public Health, under the supervision of HBV and SLR. Prior to data collection, ESM received training in the interview method by conducting three pilot interviews, also used to pilot-test the semi-structured interview guide. The interview guide was used to ensure consistency across participants, but the format remained flexible to allow for in-depth follow-up questions and exploration of participants’ individual perspectives. All interviews were conducted face-to-face in a private room at one of the pain centers. Each interview lasted approximately 30 min and was audio recorded. Participants were informed about the study’s purpose and that ESM was a master’s student.

Data for this study were collected concurrently with a study investigating the validity of PROMIS-10 GH (in review) using a modified version of the Three-Step Test Interview (TSTI) method [28]. The TSTI method is a cognitive interview technique aimed at identifying how participants interpret, and respond to questionnaire items, thereby uncovering potential issues with validity. It consists of three steps: (1) observing participants as they complete the questionnaire while thinking aloud, (2) follow-up probing to clarify responses or behaviors, and (3) a debriefing to explore their interpretations. To address the aims of this study, a fourth step was added to the TSTI method. In this step, participants were asked to revisit each item and reflect on how much their response would need to change for them to perceive it as important. The participants’ original answer from the first step of the TSTI served as the initial starting point. For items rated on the 5-point Likert scale, participants were asked to indicate how many response categories (e.g., from “fair” to “good”) would need to change for the difference to be perceived as important. For item 7r (pain intensity), rated on an 11-point numeric rating scale (0–10), participants were asked how many points their response would need to change for it to be considered important. Some participants reported a change between two categories or between two points (e.g., 1.5 or 2.5), which they were allowed to do. To support participants in reflecting on what constitutes an important change and to study baseline dependency, two hypothetical scenarios were introduced: one in which their original response was imagined to be one category or point higher (better), and one in which it was one category or point lower (worse). In each case, participants were asked again to consider how much their response would need to change - relative to the new starting point - to be perceived as important. This allowed us to assess whether the magnitude of important change is consistent across baseline levels or depends on the starting point on the response scale (Table 1).

Following each rating, participants were asked to explain why and how such a change in their response to the specific item would be important to them, including how it might affect for example their physical health, quality of life, social participation, or experience of pain intensity. They were encouraged to provide specific examples from their own lives and to reflect on what such a change could lead to in practice in terms of everyday activities and broader impacts on their daily life. Although offered, no participants reviewed the transcripts.

### Data analysis

To address the first objective, we calculated the mean change score for what participants considered an important change in their response to each PROMIS-10 GH item based on their original responses. For each item, the reported change scores were summed and divided by the number of responses to generate a mean of perceived important change alongside with 95% confidence intervals (CI). We also calculated median and interquartile range (IQR) for each item to account for potential skewness. These results are presented in Table 2. Additionally, the mean, 95% CI, median, and IQR were calculated for the two hypothetical scenarios - one in which the original response was imagined to be one category or point lower, and one in which it was one category or point higher. To further examine baseline dependency in original responses for the pain item, participants were divided into two groups based on their baseline pain severity: Baseline severity from 5 to 7 (low/moderate, *n* = 8) and baseline severity from 8 to 10 (high, *n* = 6). The mean, 95% CI, median, and IQR were calculated for the two groups. These supplementary analyses of hypothetical scenarios and baseline dependency for the pain intensity item are presented in Online Resource S1, S2, and S3. Calculations were conducted using Stata/BE 19.

To address the second objective, which explored participants’ explanations for why and how such changes in their responses to each item would be important, thematic analysis was conducted by ESM following Braun and Clarke’s six-phase approach [29]. The analysis was conducted separately for each item to capture item-specific themes, but the process followed the same six steps across all items. In step one, ESM became thoroughly familiar with data by reading and re-reading the interview transcripts and making initial notes to identify relevant content regarding participants’ reasoning and experiences. In step two, initial codes were generated by systematically identifying and labelling all data segments where participants explained why and how they perceived a given response change to be important. In step three, ESM searched for cross-code themes by organizing related codes into broader patterns that captured the underlying aspects in the participants’ responses. In step four, these preliminary themes were reviewed and refined to ensure coherence and alignment with both the coded data and the overall dataset. Step five involved defining and naming the final themes, ensuring that each theme clearly reflected the essence of participants’ perspectives and explanations. Finally, in step six, the findings were documented, and illustrative quotes were selected and translated to English to support each theme [29]. After conducting the thematic analysis, we explored whether participants perceived some response changes as more meaningful than others - for example, whether an improvement from “poor” to “fair” was considered more important than a similar change from “good” to “very good”.

## Results

### Recruitment and participants

A total of 19 individuals were recruited, of whom 17 participated in study. Fourteen interviews were conducted at the pain center at Odense University Hospital, while the remaining three took place at the pain center at the University Hospital of Southern Denmark. Three participants were accompanied by a relative, who remained present during the interview for support. Among the 17 participants, 13 were women, and participants had a mean age of 50.41 years. On a 0–10 pain intensity scale reflecting the past seven days, the mean pain intensity was 7.65. Participants reported pain in various body locations. Their mean PROMIS-10 GH T-scores were 32.19 (corresponding to poor) for physical health and 40.74 for mental health (corresponding to fair). See Table 1 for participant characteristics. 

**Table 1:** Descriptive characteristics of patients with high-impact chronic pain (*n* = 17)

Variables	Total (*n* = 17)
Sex female (n)	13
Age in years (mean (range))	50.41 (27–68)
Ethnicity (n)	
Danish	16
Other	1
Pain Intensity from 0–10 in the last 7 days (mean (range))	7.65 (5–10)
Pain location (n)	
Local (ankle, hip)	2
Regional (spine, leg, arm, head)	10
Widespread (> 1 regions)	5
PROMIS-10 Global Health scores (mean (range))	
Raw score physical health	8.35 (5–12)
Raw score mental health	10.29 (6–15)
T-score physical health	32.19 (19.9–39.8)
T-score mental health	40.74 (28.4–50.8)

### Important change in PROMIS GH

The mean change scores perceived as important for each item derived from original responses ranged from 1.09 (median = 1) for item 1 (general health) to 1.84 (median = 2) for item 8 (fatigue) for the items rated on a 5-point Likert scale. For item 7r (pain intensity), rated on a numeric scale from 0 (no pain) to 10 (worst imaginable pain), the mean important change score was 4.36 (median = 4.25). Table 2 presents a detailed overview of each PROMIS-10 GH item alongside the mean (95% CI), median (IQR), and range of changes perceived as important calculated from original responses. Further, across all items, participants required larger changes to perceive improvement important when starting from lower baseline scores, indicating baseline dependency. For pain intensity, the perceived important change was smaller in both hypothetical scenarios compared to the original responses (S1 and S2). Also, the results showed that baseline pain severity influenced the amount of change required to be perceived as important for item 7r (pain intensity). Participants reporting high baseline pain (8–10) required a mean improvement of 6.16, whereas those reporting lower baseline pain (5–7) required a mean improvement of 3.00. 

**Table 2:** Important change in PROMIS-10 global health items among patients with high-impact chronic pain (*n* = 17) calculated from original responses. The table presents each PROMIS-10 global health item alongside the mean, 95% CI, median, IQR and range of important changes. Missing responses in each item is also reported.

Item	Mean (95% CI)	Median (IQR)	Range	Missing responses
Item 1 (general health)	1.09 (0.9;1.28)	1 (0)	0.5-2	0
Item 2 (quality of life)	1.15 (0.76:1.53)	1 (1)	0–2	0
Item 3 (physical health)	1.56 (1.22;1.90)	2 (1)	0.5-3	0
Item 4 (mental health)	1.38 (0.83;1.93)	1 (1)	0–4	0
Item 5 (social activities and relationships)	1.38 (0.94;1.82)	1.5 (1)	0–3	0
Item 9r (social activities and roles)	1.5 (1.02;1.98)	1 (1)	0–3	2
Item 6 (everyday physical activities)	1.47 (1.05;1.89)	1.5 (1)	0–3	0
Item 10r (emotional problems)	1.44 (0.96–1.92)	1 (1)	0–3	0
Item 8r (fatigue)	1.84 (1.48;2.20)	2 (0.5)	0.5-3	1
Item 7r (pain intensity)	4.36 (3.05; 5.67)	4.25 (2)	0.5-8	3

The analysis related to objective two explored why and how patients perceived changes in their responses as important and identified one to two overarching themes per item. Across items, important changes were described as improvements that enhanced daily functioning and participation. Physically, this included better function, reduced pain, increased energy, and the ability to engage in activities such as household tasks, work, and leisure activities. Mentally, participants emphasized improved mood, fewer negative emotions, and greater capacity for social interaction. Overall, these changes were perceived as contributing to better physical, mental, and social health. Table 3 provides an overview of each PROMIS-10 GH item, the number of participants (N), associated themes, and illustrative quotes.

**Table 3:** Results from thematic analysis regarding perceived important change in PROMIS-10 GH items among patients with high-impact chronic pain (*n* = 17). The table presents number of participants for whom the theme was identified (N), alongside themes and explanations supported by participant quotes.

*N*	Theme(s)	Description	Supporting Quote(s)
Item 1 (general health)			
11	Physical function and social participation	Participants described that even small improvements in their general health could importantly impact their daily lives. While they acknowledged that achieving an optimal health state was unrealistic due to chronic pain and co-existing conditions, a small change allowed for increased physical activity and mobility. This could lead to greater participation in daily tasks and fewer physical limitations.	*“Well*,* then I could do more things*,* be more active*,* both socially and for myself”* (P13). *“Well*,* it would bring greater satisfaction in everyday life*,* with the things I can participate in and do at home”* (P15).
Item 2 (quality of life)			
11	Psychological well-being	Quality of life was found to be one of the most important items to improve. Participants emphasized that improvements in mental well-being, such as increased energy and reduced fatigue, were essential for enhancing overall quality of life. Even small steps toward psychological improvement were perceived as meaningful, particularly when associated with fewer negative emotions like disappointment and worry.	*”Just less worried”* (P18).
5	Participation in everyday and social life	Some participants stated that improving their quality of life would mean having the ability to engage more in daily routines and social activities. Participants described the change as important as they could take more part in hobbies and social events to maintain a sense of normalcy and well-being.	*“Just doing something*,* being creative*,* going to some kind of hobby group or workshop*,* or something like that. Those kinds of things*,* having those leisure activities that everyone else has”* (P9).
Item 3 (physical health)			
17	Physical freedom and energy	Patients emphasized that even small improvements in physical health could significantly impact their daily lives and overall well-being. Enhancements such as being able to walk longer distances, lift more, gardening or engage more in physical activity provided greater freedom and fewer limitations. Many participants indicated that these improvements would allow for more energy to engage in daily life.	*“(…) so just a small change here*,* it would mean that I could walk or lift*,* or lift*,* which I know I won’t be able to do*,* but anyway. If I could walk a little further*,* that would be lovely”* (P3).
Item 4 (mental health)			
13	Small and large improvements in mental health	For some, mental health was one of the most important items to improve. Participants linked improvements in mental health to better mood, more energy, clearer thinking, and greater social and physical engagement. For some, a large improvement was essential, while others found even small changes highly important, as it reduced limitations, enhanced coping, and improved overall well-being.	*“Well*,* I still think that’s the most crucial thing*,* it’s me*,* my psyche*,* and my well-being. That’s the most important thing*,* that I feel good*,* right?”* (P12).
14	Accepting limitations and valuing realistic change	For some participants, it was important to set realistic goals for their mental health. They described “excellent” psychological health as unrealistic, noting that even brief moments of frustration or low mood could lower their rating. Some felt that their current psychological health was already good and saw little room for further improvement.	*“I don’t think I can go higher because I don’t believe “excellent” is an option*,* so it’s unrealistic*,* I think it’s unrealistic”* (P10).
Item 5 (social activities and relationships)			
9	Energy and emotional capacity	Some participants described that even small improvements in this domain could have an important impact by increasing their energy and emotional capacity to be present with others, especially close family members. Gaining more energy was seen as crucial for being able to engage socially and emotionally, such as being able to listen, be attentive, and avoid cancelling social plans.	*“If I had a bit more energy and mental capacity*,* I would increase my social relationships”* (P10).
6	Flexibility and freedom in social participation	Improvement in this domain was also linked to having greater freedom and flexibility in social life. Participants emphasized the importance of being able to take part in social activities without the need for strict planning to conserve energy. An important change would allow for more spontaneous participation in shared physical and social activities, such as cycling or exercising with others.	*“You would not have to think about it*,* if someone came and said*,* ‘Do you want to come to the concert?’*,* then I could let go of some of those things that make me hold back”* (P2).
Item 9r (social activities and roles)			
12	Control over daily responsibilities	Participants described improvements in their ability to manage household tasks, work, and practical daily activities as highly meaningful. Being able to do the laundry, empty the dishwasher, or maintain their home independently were seen as key indicators of improvement. Some hoped to return to work or better manage their current workload. The ability to engage in personally meaningful activities, such as attending sewing or exercise groups, was closely tied to feeling in control in everyday life.	*“Well*,* I do miss my work role*,* I think … and there are also some of those everyday activities that could improve”* (P16). *“Because it would be great to have a daily life that works*,* to take part in what you feel like*,* to manage your home*,* yes*,* that would be wonderful”* (P15).
5	Being present in social and family roles	For some participants, being able to fulfill roles within their family and social circles was important. An important change was perceived to be more available for their children, supporting their partners, or being a reliable friend. Improvements in this area were associated with greater energy and capacity to say yes to social invitations, help others, and be emotionally present.	*“Well*,* just having that bit more energy to listen to what my kids are saying*,* even when they’re really noisy”* (P18).
Item 6 (everyday physical activities)			
12	Managing daily physical tasks with less pain and fatigue	Participants highlighted the importance of being able to engage in more physical activities throughout the day without being overwhelmed by pain or fatigue. Even small improvements were seen as highly valuable, as they would enable participants to cook, clean, shop, and engage in light exercise, such as walking or gardening. An important change also included the ability to complete multiple tasks in one day without needing extended rest afterward.	*“Yes*,* then I would think that I could take care of our house*,* do a bit in the garden*,* and yes*,* go for slightly longer walks without it being too much*,* and*,* well*,* those everyday things*,* yes”* (P6).
9	Independence in physical daily life	Some participants perceived an important change as being able to manage their daily physical activities independently. Participants expressed a wish to no longer rely on partners, family, or friends for help with everyday tasks. Being able to do things on their own without scheduling around someone else’s availability was associated with a greater sense of control. Improvements in this area would allow participants to go grocery shopping, attend training sessions, or care for their home and garden on their own terms.	*“Because then I would be completely independent*,* and I wouldn’t be limited either*,* because that’s who I was*,* I could do everything myself*,* I’ve always managed on my own”* (P2).
Item 10r (emotional problems)			
14	Emotional and mental relief	Participants expressed a wish to feel less emotionally burdened by anxiety, depressive thoughts, and irritability. Even small improvements were seen as important. A change would bring more peace of mind, energy, and mental clarity both for their own well-being and to be more present for others. Emotional improvement was also seen as key in coping with chronic pain.	*“Well*,* I don’t want to go around feeling depressed or irritable neither for my own sake nor for the sake of those around me”* (P10).
Item 8r (fatigue)			
8	Physical energy and everyday functioning	Some participants found that fatigue was one of the most important items to improve. Change in physical energy was important to make it easier to carry out daily physical activities without constantly being held back by fatigue. Participants emphasized that feeling more rested would enable them to be more physically active and complete chores without becoming exhausted.	*“It would ease the system if I wasn’t so tired*,* I’d be able to do more”* (P3).
12	Mental energy and emotional stability	Some participants stated that less fatigue was important as it would lead to greater mental energy and emotional stability. Participants described how fatigue impacted their mood, causing them to be irritable and mentally drained. With more energy, they could see things in a brighter light, engage more social, and enjoy time with family and friends.	*“A lighter mindset in some way*,* because fatigue pulls you the other way*,* but when you’re less tired*,* you see things in a brighter light”* (P9).
Item 7r (pain intensity)			
7	Physical function and activities	Pain was perceived as one of the most important items to improve. A reduction in pain intensity was perceived as essential for improving physical function and enabling participation in everyday activities. Participants described how the change would allow them to move more freely, increase their level of physical activity, and engage in tasks currently limited by pain. Reduced pain was thus associated with greater independence, energy, and the ability to pursue meaningful physical goals. For many participants, a substantial reduction in pain was considered important. Minor improvements were often seen as insufficient, as they would not importantly change daily life.	*“If I could get down to that level*,* many things would be resolved. Then I could start cycling again*,* I could be more physically active*,* and I would have more energy for all the things I want to do” (P19).* *“No pain – that would truly be wonderful. I feel like I’ve already been through enough*,* so why should I also have to live with pain?” (P12).*
8	Psychological well-being	Participants linked pain relief to improved psychological well-being, including better sleep, enhanced concentration, and greater emotional stability. Pain was often described as a persistent cognitive and emotional burden. Lowering pain levels was seen as a prerequisite for regaining mental clarity, sustaining attention, and engaging more fully in social and cognitive activities.	*“I’m struggling with these cognitive challenges. It’s frustrating that I lose words and can’t remember things. I would like to have the energy to participate again*,* without it taking me several days to recover afterwards” (P16).*

The qualitative analysis also showed that participants generally viewed moving from, for example, “poor” to “fair” as more meaningful than making the same categorical improvement from “good” to “very good” across items since progress from a poor state was considered to have a greater impact on daily life. For some, this was because being at the “good” or “very good” level already reflected a state of satisfaction, where further improvement was perceived as less significant as seen in the following quote: *“Who would really strive for much more than ‘good’?”* (P1) (item 1, general health). Further, some participants considered changes from 4 to 5 as unrealistic, especially concerning item 4 on mental health: “*I don’t think I can go higher because I don’t believe ‘excellent’ is an option*,* so it’s unrealistic*,* I think it’s unrealistic”* (P10). Others reported that no further improvement was necessary because they were already content with their current state.

## Discussion

### Summary of findings

The analysis related to objective one showed that, for items rated on the 5-point Likert scale, the mean change score perceived as important ranged from 1.09 (median = 1) for item 1 (general health) to 1.84 (median = 2) for item 8r (fatigue). For item 7r (pain intensity), rated on the 11-point numeric rating scale (0–10), participants indicated that a mean change of 4.36 (median = 4.25) was required for the change in their response to be considered important. The thematic analysis addressing objective two identified one to two themes per item, capturing participants’ reflections on why and how a change in response to a specific item was perceived as important, and how such change would manifest in their daily lives. These perspectives reflected varied lived experiences and anticipated improvements across physical, psychological, and social domains of health.

### Discussion of findings

Direct comparisons with existing research are limited, as no qualitative studies have explored important change in PROMIS-10 GH items, either generally or in chronic pain populations. Previous research has primarily used quantitative methods to estimate MIC thresholds at the domain level, with anchor-based approaches offering the most patient-relevant results [16]. In knee arthroplasty populations, MIC thresholds of 2.5–6.59 T-score units for physical and 1.64 T-score units for mental health have been reported [10, 11]. Interpreted within the general PROMIS-10 T-score ranges (16.2–67.7 for physical and 21.2–67.6 for mental health [23]), these correspond approximately to raw score changes of 1–2 and 0.5-1 points, respectively. The physical health scores are broadly consistent with the results in the present study, whereas the mental health scores are slightly lower than our results. While such quantitative estimates are informative, this study extends current knowledge by providing qualitative insights into what constitutes important change for patients at item-level, why it matters, and how it affects their everyday lives - insights that are increasingly valuable as item-level thresholds are used to guide clinical dialogue and patient-centered care [13].

A study by Kitchen et al. [30] explored perceived meaningful change among patients with endometriosis using the Endometriosis Symptom Diary (ESD) and Endometriosis Impact Scale (EIS). Although not directly comparable with PROMIS-10 GH, these PROMs address overlapping domains such as pain, emotional well-being, and functioning. In the study by Kitchen et al. [30], patients considered both actual and hypothetical changes in “worst pelvic pain” in the ESD on a 0–10 pain intensity scale, with most patients identifying a 2-3-point reduction as meaningful, depending on baseline severity. In the present study, participants considered a mean of 4.36-point reduction (median = 4.25) in their response on the 0–10 pain scale to be important, reflecting a need for greater change. Reasons for this were similar, e.g. experiencing less pain, improved social participation, and physical/daily activities, but also extended further to include enhanced psychological well-being. Further, quantitative studies have reported that a 2.17-point reduction on the 0–10 NRS is meaningful in shoulder pain [31], and that 1-2-point reductions represent slight to substantial improvement in chronic musculoskeletal pain [32]. In contrast, participants in the present study indicated that a larger reduction in pain intensity was needed. This may reflect the fact that participants in this study had higher baseline pain intensity than average. This is supported by a systematic review showing that absolute MIC differences are strongly associated with baseline pain intensity [33]. Also, the study by Kitchen et al. [30] found that patients perceived greater improvement as meaningful when baseline pain levels were higher. The present study showed similar baseline dependency across all PROMIS-10 GH items rated on the 5-point Likert scale. Results from the hypothetical scenarios (S1 and S2) indicated that starting one category lower than the original response required larger changes in their responses to be perceived as important, whereas starting one category higher required smaller changes. For the pain intensity item, starting one point higher on the 0–10 scale required a greater change in their response (mean = 4.27) than starting one point lower (mean = 4.00), but notably the important change score derived from original responses (mean = 4.36) was slightly higher than both. Further, when participants were divided into two groups by baseline pain severity, those reporting higher baseline pain intensity required substantially greater improvement in their response (mean = 6.16) than those with lower pain (mean = 3.00) (S3). Also, the participants qualitative accounts showed that moving from “poor” to “fair” was perceived to be more meaningful than an equivalent improvement from “good” to “very good”. Collectively, these findings support the notion of baseline dependency in perceived important change.

The study by Kitchen et al. [30] explored which EIS domains patients found most important to improve and why, identifying two primary domains: emotional well-being and physical functioning. Emotional well-being was prioritized due to the impact of chronic pelvic pain on mood, with patients reporting irritability, sadness, and social withdrawal, which hindered daily life and relationships. This aligns with the present study, where mental health was frequently mentioned as a key domain to improve, as participants associated better mental health with improved mood, energy, clarity, and capacity for social and physical engagement. Physical functioning was also rated highly important by Kitchen et al. [30], as reduced capacity - such as being unable to exercise, play with children, or sit through a film - disrupted their sense of normalcy and independence. Similarly, participants in the present study described that even small improvements in physical function, such as walking longer distances, lifting objects, or engaging in activities with less pain, were perceived as meaningful. Finally, Kitchen et al. [30] noted that broader limitations, including sleep difficulties, inability to work, and reduced social engagement, were also important. In the present study, fatigue was particularly emphasized as a concern affecting both physical and mental energy and sustaining social roles and improving quality of life were also considered important areas.

### Strengths, limitations, and methodological reflections

#### Participant selection and characteristics

Participants were purposively sampled to ensure variation in age, gender, and ethnicity [34], thereby strengthening the study’s information power [35]. The sample reflected key traits of the wider population of patients with HICP, which is primarily composed of middle-aged women, but participants reported slightly poorer physical and somewhat better mental well-being than the general HICP population in Denmark [2]. Limited ethnic diversity (only one participant of non-Danish background) may constrain transferability to more diverse populations [36]. Recruitment through PainData may also have introduced selection bias [37], as individuals with fewer cognitive or personal resources may have been less likely to participate due to the demanding questionnaire.

#### Data collection

The data collection design aligned with the study’s aims. A key strength was the flexible semi-structured interview format, which ensured consistency while allowing participants to elaborate on their experiences and illustrate what important change meant in daily life. The use of the TSTI method further strengthened the approach, as participants’ familiarity with the PROMIS-10 GH items facilitated reflection. Combining original and hypothetical scenarios also helped participants consider change from multiple perspectives.

However, several limitations should be noted. The interview approach was not based on a validated method for assessing item-level important changes, limiting comparability and reproducibility. The interviews were long and cognitively demanding, which may have caused fatigue and affected data quality. Participants’ reflections may also have been influenced by the preceding TSTI interview, introducing response bias [38]. Finally, the hypothetical scenarios were challenging for some, particularly when imagining improvement, which is reflected in the missing responses (S1 and S2).

#### Data analysis

The quantitative analysis of item-level changes of responses offered an overview of mean important change scores but limited comparability, as most studies report changes at domain or T-score level. The thematic analysis, following Braun and Clarke’s framework [29], provided richer insights into participants’ reasoning and everyday contexts, complementing quantitative findings. Although the analytic process was systematic and reflective, it was only conducted by ESM, which may have introduced researcher bias [39]. However, the findings were discussed within the research team.

### Conclusion and future directions

This study is the first to qualitatively examine perceived important changes in responses to items in PROMIS-10 GH among individuals with HICP. For 5-point Likert items, the mean of important change in responses ranged from 1.09 (item 1, general health) to 1.84 (item 8r, fatigue). For pain intensity (item 7r, 0–10 scale), the mean important change was 4.36. The thematic analysis identified one to two themes per item, reflecting expected improvements across physical, mental, and social domains. While findings provide novel insights into patient-defined important changes, the small sample and specific settings of the pain centers limit generalizability. Further research should focus on item-level important change in PROMIS-10 GH across broader populations and refine methodologies for capturing what constitutes important change from the patient’s perspective.

## Supplementary Information

Below is the link to the electronic supplementary material.

Supplementary Material 1

## Data Availability

The datasets generated and analyzed during the current study are not publicly available due to privacy regulations.

## Code availability

Not applicable. No custom code or software was used in this study.

## Declarations

### Data protection

The study followed the General Data Protection Regulation (GDPR) regarding the legal and secure handling of personal data. All data were pseudonymized, securely stored, and access was restricted to the research team.
