# Supplementary material for: What constitutes important change in individual PROMIS-10 global health items in patients with high-impact chronic pain: a qualitative interview study
Source: Qual Life Res. 2026 Jan 22;35(2):50. doi: 10.1007/s11136-026-04164-5 (PMC12827400; doi:10.1007/s11136-026-04164-5)
Supplement: Supplementary file 1 — Supplementary Material 1 [file 11136_2026_4164_MOESM1_ESM.docx]

**ONLINE RESSOURCE**

**Article title:** What constitutes an important change in individual PROMIS-10 Global Health items in patients with high-impact chronic pain - A qualitative interview study

**Journal name:** Quality of Life Research

**Author names:** Emily Sophia Madley, Daniel Broholm, Sophie Lykkegaard Ravn and Henrik Bjarke Vaegter

**Affiliation first author:** Odense University Hospital

**Email first author**: [emilysophiamadley@gmail.com](mailto:emilysophiamadley@gmail.com)

**S1:** *Important change in PROMIS-10 Global Health items among patients with high-impact chronic pain (n=17) calculated from hypothetical response starting one point or category lower than the original response. The table presents each PROMIS-10 Global Health item alongside the mean, 95% CI, median, IQR and range of important changes. Missing responses in each item is also reported.*

| **Item** | **Mean (95% CI)** | **Median (IQR)** | **Range** | **Missing responses** |
| --- | --- | --- | --- | --- |
| Item 1 (general health) | 1.37 (1.00;1.74) | 1 (1) | 0.5-3 | 2 |
| Item 2 (quality of life) | 1.66 (1.21;2.10) | 1.5 (1) | 0.5-3 | 1 |
| Item 3 (physical health) | 1.81 (1.18;2.43) | 2 (1) | 0.5-4 | 4 |
| Item 4 (mental health) | 1.62 (1.22;2.01) | 2 (1) | 1-3 | 4 |
| Item 5 (social activities and relationships) | 1.96 (1.38;2.54) | 2 (2) | 1-4 | 3 |
| Item 9r (social activities and roles) | 1.96 (1.24;2.69) | 2 (2) | 0-4 | 4 |
| Item 6 (everyday physical activities) | 1.79 (1.37;2.21) | 2 (1) | 1-3 | 3 |
| Item 10r (emotional problems) | 1.69 (1.30;2.01) | 1.75 (1) | 1-3 | 1 |
| Item 8r (fatigue) | 1.8 (1.32;2.28) | 2 (1.5) | 0.5-3 | 2 |
| Item 7r (pain intensity) | 4 (2.07;5.92) | 3.5 (2.5) | 1-10 | 7 |

**S2:** *Important change in PROMIS-10 Global Health items among patients with high-impact chronic pain (n=17) calculated from hypothetical response starting one point or category higher than the original response. The table presents each PROMIS-10 Global Health item alongside the mean, 95% CI, median, IQR and range of important changes. Missing responses in each item is also reported.*

| **Item** | **Mean (95% CI)** | **Median (IQR)** | **Range** | **Missing responses** |
| --- | --- | --- | --- | --- |
| Item 1 (general health) | 0.84 (0.65;1.03) | 1 (0) | 0-1 | 1 |
| Item 2 (quality of life) | 0.94 (0.82;1.07) | 1 (0) | 0.5-1 | 8 |
| Item 3 (physical health) | 0.96 (0.7;1.22) | 1 (0) | 0-2 | 4 |
| Item 4 (mental health) | 1.29 (0.83;1.75) | 1 (0.75) | 0.5-3 | 5 |
| Item 5 (social activities and relationships) | 1.17 (0.74;1.60) | 1 (0) | 1-2 | 11 |
| Item 9r (social activities and roles) | 0.91 (0.46;1.36) | 1 (0.5) | 0-2 | 6 |
| Item 6 (everyday physical activities) | 0.9 (0.50;1.30) | 1 (0) | 0-2 | 7 |
| Item 10r (emotional problems) | 1.09 (0.47;1.71) | 1 (1.5) | 0-3 | 6 |
| Item 8r (fatigue) | 1.5 (0.97;2.03) | 1.25 (1) | 0.5-3 | 7 |
| Item 7r (pain intensity) | 4.27 (3.10;5.44) | 4 (3) | 2-7 | 6 |

**S3:** *Important change for item 7r (pain intensity) in PROMIS-10 Global Health across two baseline severity groups on the 0-10 pain intensity scale, illustrating baseline dependency. The supplementary table presents each group (baseline pain intensity between 5-7 and baseline pain intensity between 8-10) alongside the number of participants in each group, mean, 95% CI, median, IQR and range of important changes.*

| **Baseline pain severity group** | **Number of participants in the group** | **Mean (95% CI)** | **Median (IQR)** | **Range of important change** |
| --- | --- | --- | --- | --- |
| 5-7 | 8 | 3.00 (1.68; 4.32) | 3 (2.25) | 0.5-5 |
| 8-10 | 6 | 6.16 (4.36, 7.97) | 6 (3) | 4-8 |
